# How do patients and physicians perceive immune thrombocytopenia (ITP) as a disease? Results from Indian analysis of ITP World Impact Survey (I-WISh)

**DOI:** 10.1186/s41687-022-00429-y

**Published:** 2022-03-18

**Authors:** Prantar Chakrabarti, Biju George, Chandrakala Shanmukhaiah, Lalit Mohan Sharma, Shashank Udupi, Waleed Ghanima

**Affiliations:** 1 Department of Hematology, Vivekananda Institute of Medical Sciences, Machan, L 16 Panchasayar, Kolkata, West Bengal 700094 India; 2 Department of Hematology, CMC Vellore, Vellore, Tamil Nadu 632004 India; 3 Department of Clinical Hematology, KEM Hospital, 1902, 19th floor UG PG hostel, KEM Hospital Campus, Parel, Mumbai, Maharashtra 400012 India; 4 Department of Medical Oncology, MG Medical College, 67/166, Sector 6, Pratap Nagar, Jaipur, Rajasthan India; 5 Medical Affairs, Oncology (Hematology), Novartis Healthcare Private Limited, Inspire BKC, Part of 601 & 701, 7th Floor, Bandra Kurla Complex, Bandra (East), Mumbai, Maharashtra 400051 India; 6 Departments of Research and Hemato-Oncology, Østfold Hospital, Østfold Hospital, PB 300, 1714 Grålum, Norway

**Keywords:** Disease management, Health-related quality of life (HRQoL), Immune thrombocytopenia (ITP), India, ITP World Impact Survey (I-WISh), ITP symptoms

## Abstract

**Purpose:**

Immune thrombocytopenia (ITP) is primarily considered a bleeding disorder; its impact on patients’ health-related quality of life (HRQoL) is under-recognized. We aimed to assess how aligned patient and physician perceptions are regarding ITP-associated symptoms, HRQoL, and disease management in India.

**Methods:**

Patients and physicians (hematologists/hemato-oncologists) from India who participated in the global ITP World Impact Survey (I-WISh) were included in this subgroup analysis (survey). Physicians were recruited via a local, third party recruiter in India. In addition to completing a survey themselves, physicians were asked to invite consulting patients on a consecutive basis to complete a survey. All surveys were completely independently by the respondents online in English. The respondents took 30 min to complete the questionnaire. Patients also completed the newly developed ITP Life Quality Index (ILQI) that included 10 questions on the impact of ITP on the following: work or studies, time taken off work or education, ability to concentrate, social life, sex life, energy levels, ability to undertake daily tasks, ability to provide support, hobbies, and capacity to exercise.

**Results:**

A total of 65 patients and 21 physicians were included in this study. Average disease duration from diagnosis-to-survey-completion was 5.3 years. The most severe symptoms reported by patients at diagnosis were menorrhagia (15 of 19 patients [79%]), anxiety surrounding unstable platelet counts (17 of 28 patients [61%]), and fatigue (27 of 46 patients [59%]); these were also the key symptoms they wanted to be resolved. In contrast, physicians perceived petechiae (19 of 21 patients [90%]), bleeding-from-gums (8 of 21 patients [86%]), and purpura (16 of 21 patients [76%]) as the most common symptoms. While the important treatment goals for patients were healthy blood counts (42 of 65 patients [65%]), improved QoL (35 of 65 patients [54%]), and prevention of worsening of ITP (33 of 65 patients [51%]), physicians’ goals were reduction in spontaneous bleeding (17 of 21 physicians [81%]), better QoL (14 of 21 physicians [67%]), and symptom improvement (9 of 21 physicians [43%]). More than half the patients reported that ITP affected their work life/studies, social life, and energy levels, thereby negatively impacting their QoL. Patients were almost entirely dependent on family and friends for support.

**Conclusions:**

This survey highlights the substantial discrepancy in patients’ and physicians’ perceptions regarding ITP-associated symptoms and treatment goals in India. Based on the identified gaps, educating physicians on aspects of ITP beyond bleeding, and highlighting patients’ under-recognized symptoms/needs through support-systems should be prioritized in the future.

**Supplementary Information:**

The online version contains supplementary material available at 10.1186/s41687-022-00429-y.

## Background

Immune thrombocytopenia (ITP) is an acquired autoantibody-mediated bleeding disorder characterized by both accelerated platelet destruction and impaired platelet production, which an estimated incidence in adults between 1.6 and 3.9 per 100,000 person-years based on the platelet count threshold used [1]. ITP requires lifelong treatment in a substantial proportion of adult patients, thereby negatively impacting the patient quality of life (QoL) [1, 2]. Improvement in health-related QoL (HRQoL) parameters has been identified as an important treatment objective in the updated ITP guidelines (ASH, ICR 2019) [3, 4]. However, in resource-limited countries, such as India, where physicians have a higher patient burden and can afford only limited in-clinic time [5–7], assessment and treatment of HRQoL parameters is challenging. Physicians often tend to underestimate or ignore HRQoL parameters in routine clinical practice, as the major treatment goal for ITP is to treat or prevent bleeding [2].

Recently, the ITP World Impact Survey (I-WISh) was conducted to discern how ITP and associated treatments affect patient lives and to evaluate how aligned patient and physician perceptions are regarding symptoms, HRQoL, and disease management [8, 9], and we have conducted an analysis of data from the Indian patient subgroup included in the I-WISh study. With ITP being one of the most common non-infectious causes of thrombocytopenia in India [10, 11], the major objectives of this study were to understand the challenges in the diagnostic journey of patients with ITP in India; patient and physician perceptions of disease and symptoms; impact of ITP on patient QoL, daily activities, and work; and existing support systems for ITP and its management.

## Methods

### Survey participants and study conduct

The I-WISh India-specific analysis is based on data collected as part of I-WISh 1.0, a cross-sectional survey of adult patients (age ≥ 18 years) with ITP and hematologists or hemato-oncologists who treat patients with ITP. The global I-WISh study was conducted in 13 countries (Canada, China, Colombia, Egypt, France, Germany, India, Italy, Japan, Spain, Turkey, the United Kingdom, and the United States).

Patient surveys were sent via mass email to patient support networks and physicians who were requested to disseminate the surveys to patients. A steering committee of disease experts and patient advocates led the design of the survey materials and endorsed them prior to initiation of data collection. Physician surveys were emailed by local fieldwork agencies. Physicians were recruited via a local, third party recruiter in India. Physicians invited patients to complete the survey following a routine consultation for their ITP. In addition to completing a survey themselves, physicians were asked to invite consulting patients on a consecutive basis to complete a survey. All surveys were completely independently by the respondents online in English. As the surveys were online, it was not possible for any respondent to omit to answer any question. However, where deemed appropriate, respondents were allowed to select ‘Not applicable’ or ‘Don’t know’ for certain questions. In these cases, the ‘Don’t know’ or ‘Not applicable’ responses were removed from the individual analyses, but these patients were otherwise eligible for inclusion in all other analyses. Overall, the respondents took 30 min to complete the questionnaire. Fully deidentified respondent information was collated and aggregated by local fieldwork partners such that the surveys were unlinked and anonymized. Surveys and details of the survey methods, including how patients and physicians were identified, have been outlined in the supplementary material and published previously [8, 9].

To understand the level of agreement that the respondents had with a statement in the survey, a Likert scale of 1–7 was used; for assessment of symptoms, a score ≥ 5 on the Likert scale implied that the symptom in question was considered to be “severe” by the patient. Patients also completed the newly developed ITP Life Quality Index (ILQI) that included 10 questions on the impact of ITP on the following: work or studies, time taken off work or education, ability to concentrate, social life, sex life, energy levels, ability to undertake daily tasks, ability to provide support, hobbies, and capacity to exercise [12]; additional details can be accessed from the global I-WISh study [10].

All methods were carried out in compliance with EphMRA guidelines and in full accordance with the US HIPAA 1996. Separate protocols were submitted for the patient and physician surveys (patient survey reference number: 02018/1056; physician survey reference number: 02018/1049). Survey materials and protocol were reviewed and approved by the Western Institutional Review Board. Patients and physicians were given an overview of the study and ethical approval details; those who wished to participate had to provide consent via a tick/check box before initiating.

### Statistical analyses

It being a descriptive, exploratory study, it did not include sample size calculations. The local recruiter conducted a feasibility assessment prior to launching data collection to confirm the achievable sample size for this study.

Patient and physician survey data were analyzed separately using descriptive statistics. Analyses were descriptive, and no formal hypothesis was tested. Missing data were not imputed. Analyses were conducted using STATA statistical software version 15.1 (StataCorp, College Station, TX).

## Results

### Demographic characteristics and the diagnostic journey of patients with ITP

Overall, 21 physicians and 65 patients completed the survey questionnaire from March 09, 2018 and May 02, 2018. Patients were recruited by either experienced physicians treating ITP (64 of 65 patients [98%]) or patient association groups (1 of 65 patients [2%]). Accurate estimates on the number of individuals who were approached for participation in the survey could not be obtained. All respondents who participated in the survey questionnaire provided their demographic information, along with details of their diagnostic processes (Table 1).

**Table 1:** Patient/physician demographic characteristics and patient diagnostic pathways

	Patients N = 65
Mean age, years	33
Male, n (%)	39 (60%)
Female, n (%)	26 (40%)
Current health state (Score: 1, very poor health; 7, excellent health)	
≤ 4	26 (40%)
Splenectomized, n (%)	6/64 (9.3%)
**Diagnosis**	
Median (IQR) time from symptom presentation to diagnosis, months	1.5 (0.5–5.7)
Symptom presentation to first consultation, months	0.7 (0.1–3.0)
First consultation to diagnosis	0.5 (0.2–1.0)
Patients with a median time from initial presentation to ITP diagnosis > 6 months, n (%)	9/55 (16%)
Patients in whom diagnosis of ITP confirmed as a result of another health condition, n (%)	2 (3%)
Delay in diagnosis, n (%)	21 (32%)
Awaiting additional test results	8 (38%)
Specialist reference	7 (33%)
Patient support following diagnosis, n (%)	
Family/friends	59 (91%)
Physicians	50 (77%)
Nurses	24 (37%)
Patients who needed more support during the diagnosis process, n (%)	27 (42%)
Physicians	20 (74%)
Family/friends	13 (48%)
Patient support groups	11 (41%)

	Physicians N = 21
Average total patient caseload	625
Number of ITP patients seen in the last 12 months	81
Practice setting	
Private care	12 (57%)
Specialty cancer center	5 (24%)
University teaching hospital and community teaching hospital	4 (19%)
Year of qualification	
Before 1981	1 (5%)
1981–1993	3 (14%)
1994–2003	5 (24%)
2004–2014	10 (48%)
After 2014	2 (10%)
**Diagnosis**	
Median (IQR) time from symptom presentation to diagnosis, months	0.25 (0.25–0.62)
Primary ITP	70%
Secondary ITP	30%
Reasons for delay in diagnosis	
Specialist reference	13 (62%)
Exclusion of other potential causes	12 (57%)
Causes of secondary ITP	
Systemic lupus erythematosus	11 (52%)
Drug-induced thrombocytopenia	11 (52%)
Hepatitis C virus	10 (48%)
Chronic lymphocytic leukemia	8 (38%)
Human immunodeficiency virus	7 (33%)
Investigation rates (asymptomatic vs high symptom burden)	
Spleen evaluation	12 (57%) vs 16 (76%)
Coomb’s test	5 (24%) vs 13 (76%)
*H. pylori*	3 (14%) vs 8 (28%)
Computed tomography scan	1 (5%) vs 7 (33%)
Platelet specific assay	0% vs 4 (19%)
Misdiagnosis rates	
Upto 25% patients are misdiagnosed	14 (67%)
26–50% patients are misdiagnosed	5 (24%)
Most commonly misdiagnosed conditions	
Drug induced thrombocytopenia	12 (63%)
Leukemia	11 (58%)
Aplastic anemia	10 (53%)

#### Patients

The mean (standard deviation [SD]) age of the patients was 33 (12.62) years, with 39 of 65 patients (60%) being male. The symptom burden was moderate to high in 24 of 57 patients (42.1%), of whom 17 patients (71%) reported a poor health score (≤ 4 on the Likert scale). Patients met an average of 5 healthcare professionals (HCPs; including primary care physicians, nurses, emergency care doctors, dentists, and others) before an accurate diagnosis of ITP, which was confirmed by physicians specialized in the management of ITP in 56 of 65 patients (86%). Overall, 21 of 65 patients (32%) expressed a delay in ITP diagnosis, thereby leading to severe anxiety (≥ 5 on the Likert scale) in 8 of 21 patients (38%) (Table 1).

#### Physicians

All physicians included in the survey were either hematologists (n = 13) or hemato-oncologists (n = 8). More than half of the physicians who participated in the survey (12 of 21 physicians [57%]) practiced in a private setting. Of the average caseload, about 87 of 625 (13.9%) were patients with ITP. ITP was rated as a “somewhat less important” condition by 14 of 21 physicians (67%). Nearly one-fourth (5 of 21 physicians [24%]) of the physicians perceived that 26%-50% of patients were misdiagnosed (Table 1).

### Patient and physician perception of ITP symptoms and severity

#### Patient perspective

**Fig. 1:**
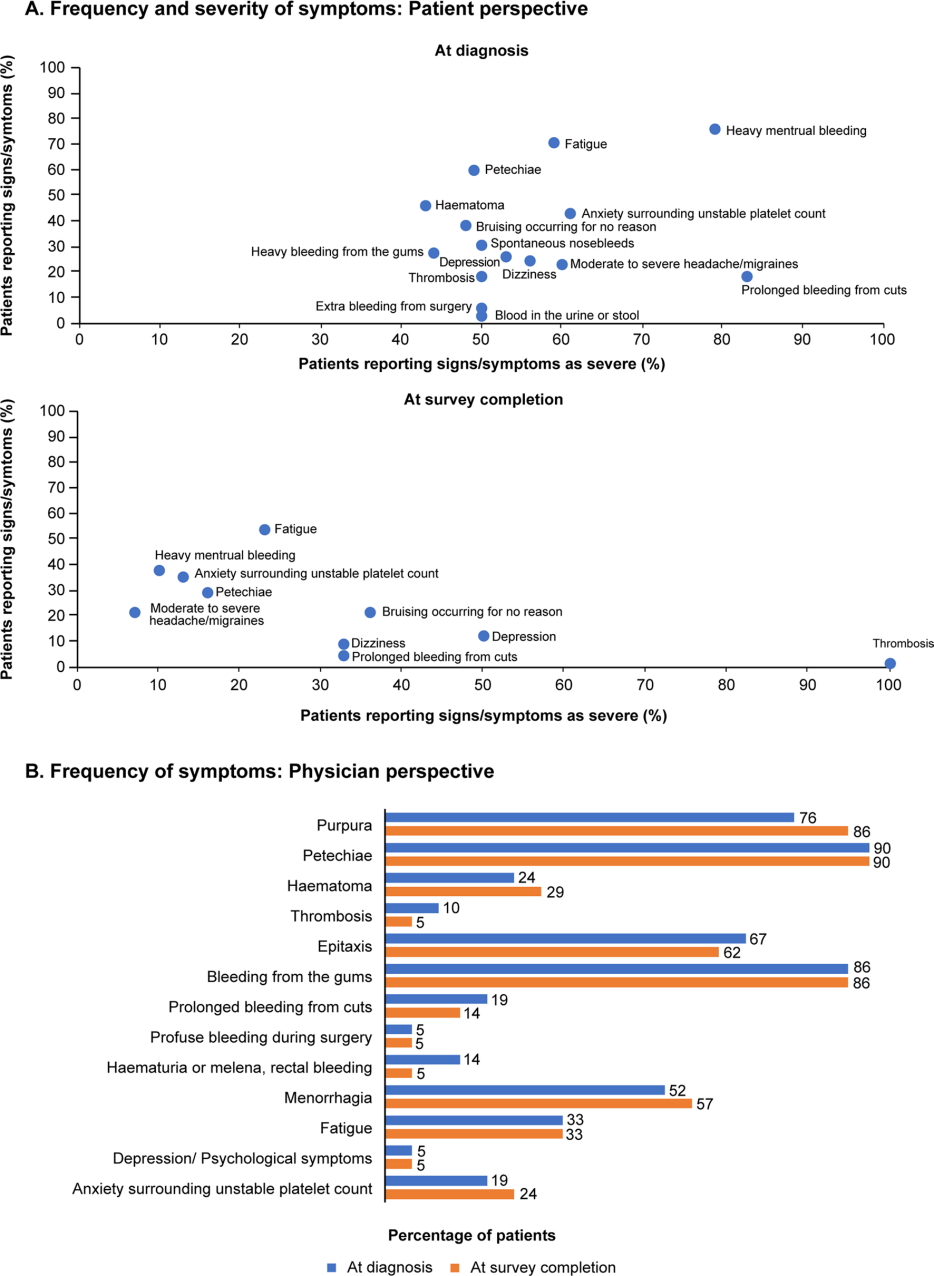
Frequency and/or severity of symptoms of ITP at diagnosis and survey completion – Patient and physician perspective

The most commonly reported symptoms at diagnosis were heavy menstrual bleeding (19 of 26 patients [73%]), fatigue (46 of 65 patients [71%]), petechiae (39 of 65 patients [60%]), hematoma (30 of 65 patients [46%]), and anxiety surrounding unstable platelet counts (28 of 65 patients [43%]). The mean (SD) duration of the disease from diagnosis to survey completion was 5.3 (6.77) years. At survey completion, the most commonly reported symptoms were fatigue (35 of 65 patients [54%]), heavy menstrual bleeding (10 of 26 patients [38%]), anxiety surrounding unstable platelet counts (23 of 65 patients [35%]), petechiae (19 of 65 patients [29%]), and hematoma (11 of 65 patients [17%]) (Fig. 1A). Menorrhagia (15 of 19 patients [79%]), anxiety surrounding unstable platelet counts (17 of 28 patients [61%]), and fatigue (27 of 46 patients [59%]) were the most commonly reported severe symptoms at diagnosis (considering symptoms reported by at least 15 patients) (Fig. 1A). The key symptoms that patients wanted to be resolved included fatigue (27 of 65 patients [42%]), heavy menstrual bleeding (10 of 26 patients [38%]), and anxiety surrounding unstable platelet counts (21 of 65 patients [32%]) (Additional file 1: Figure S1).

#### Physician perspective

The most common signs and symptoms reported by physicians, based on the inputs received from their patients, were similar at diagnosis vs survey completion; these included petechiae (19 of 21 physicians [90%] vs 19 of 21 physicians [90%]), bleeding from gums (18 of 21 physicians [86%] vs 18 of 21 physicians [86%]), purpura (16 of 21 physicians [76%] vs 18 of 21 physicians [86%]), epistaxis (14 of 21 physicians [67%] vs 13 of 21 physicians [62%]), and heavy menstrual bleed (11 of 21 physicians [52%] vs 12 of 21 physicians [57%]) (Fig. 1B). According to physicians, hematuria, melena, or rectal bleed (17 of 21 physicians [81%]); profuse bleeding during surgery (16 of 21 physicians [76%]); menorrhagia (14 of 21 physicians [67%]); anxiety surrounding unstable platelet counts (12 of 21 physicians [57%]); and hematoma (12 of 21 physicians [57%]) could have a major negative impact on patient HRQoL (scored ≥ 5 on the Likert scale). According to physicians, about 37% patients experienced fatigue, and the severity was considered as low (≤ 4 on the Likert scale) in most patients (17 of 21 physicians [81%]). Fatigue was considered to be very severe by 12 of 21 physicians (57%), 10 of 21 physicians (48%), and 8 of 21 physicians (38%) when platelet counts were <10 × 10^9^/L, 10-29 × 10^9^/L, and 30-39 × 10^9^/L, respectively (Additional file 2: Figure S2). Overall, fatigue was considered as a major concern by only 7 of 21 physicians (33%).

### Impact of ITP on QoL

#### Patient

**Fig. 2:**
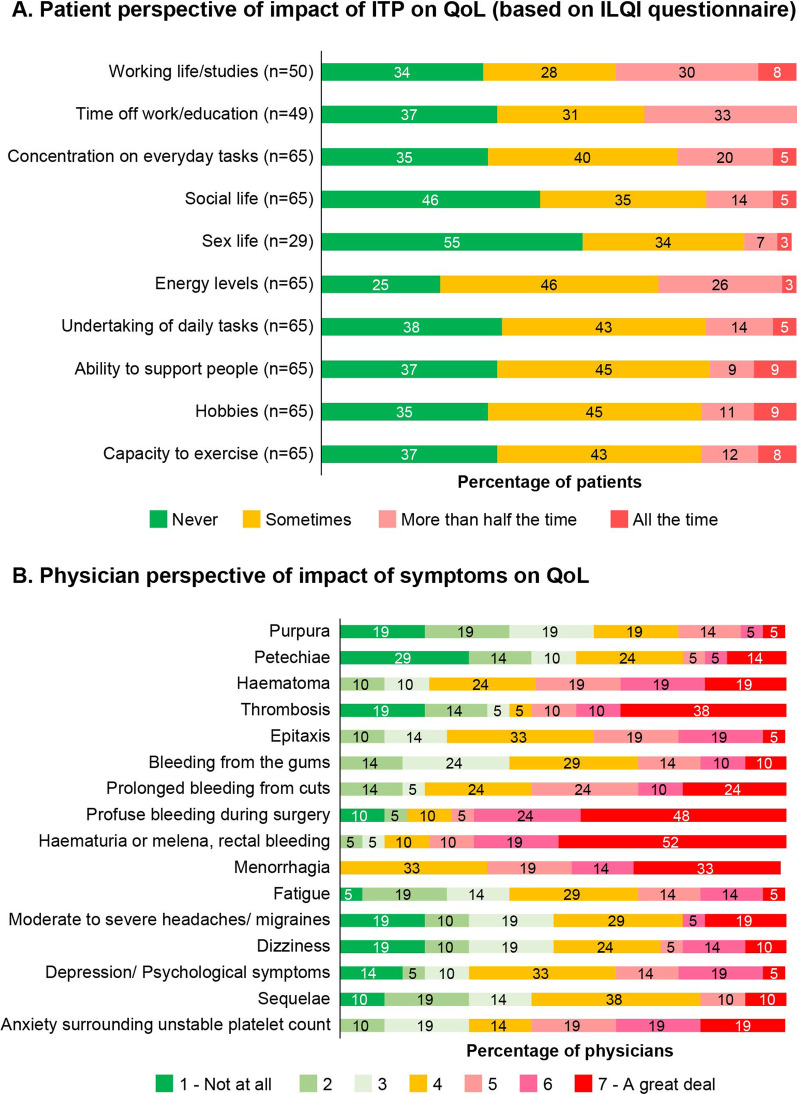
Impact of ITP on QoL – Patient and physician perspective

Based on the ILQI scores, the parameters that significantly had a negative impact on patient QoL very often (more than half of the time) were work life/studies (19 of 50 patients [38%]), absence of work/education (16 of 48 patients [33%]), and energy levels (19 of 65 patients [29%]) (Fig. 2A).

**Fig. 3:**
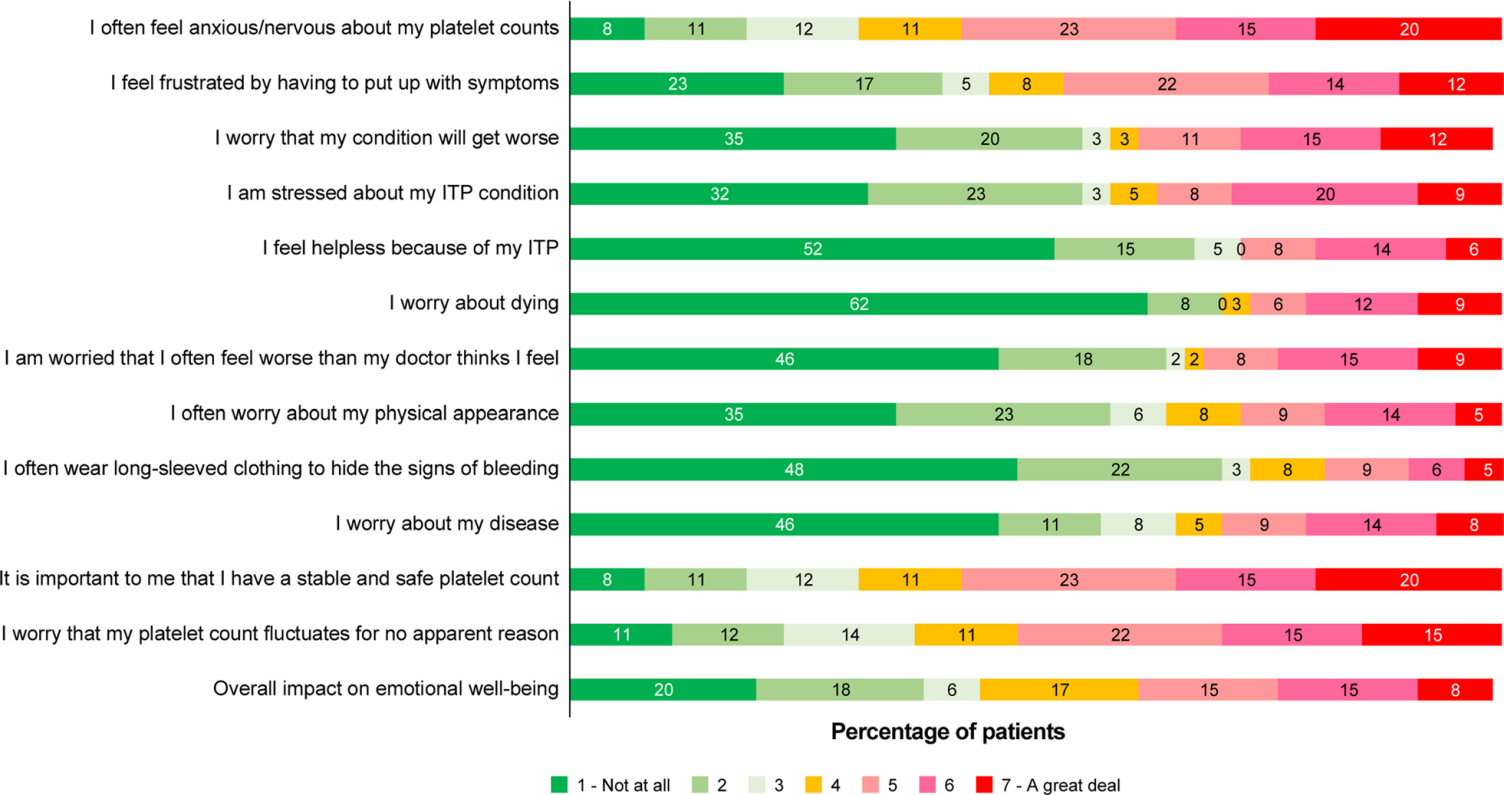
Impact of ITP on emotional well-being – Patient perspective

The overall impact on emotional well-being was scored ≥ 5 on the Likert scale by 25 of 65 patients (38%), and the top 4 reported reasons with a severe impact were anxiety surrounding unstable platelet counts (38 of 65 patients [58%]), importance of stable platelet counts (38 of 65 patients [58%]), fluctuation in platelet counts for no apparent reason (34 of 65 patients [52%]), and frustration with ITP symptoms (31 of 64 patients [48%]) (Fig. 3). Overall, 60 of 65 patients (92%) did not receive any professional support, of whom 20 patients (33%) expressed a desire for additional support (data not shown).

**Fig. 4:**
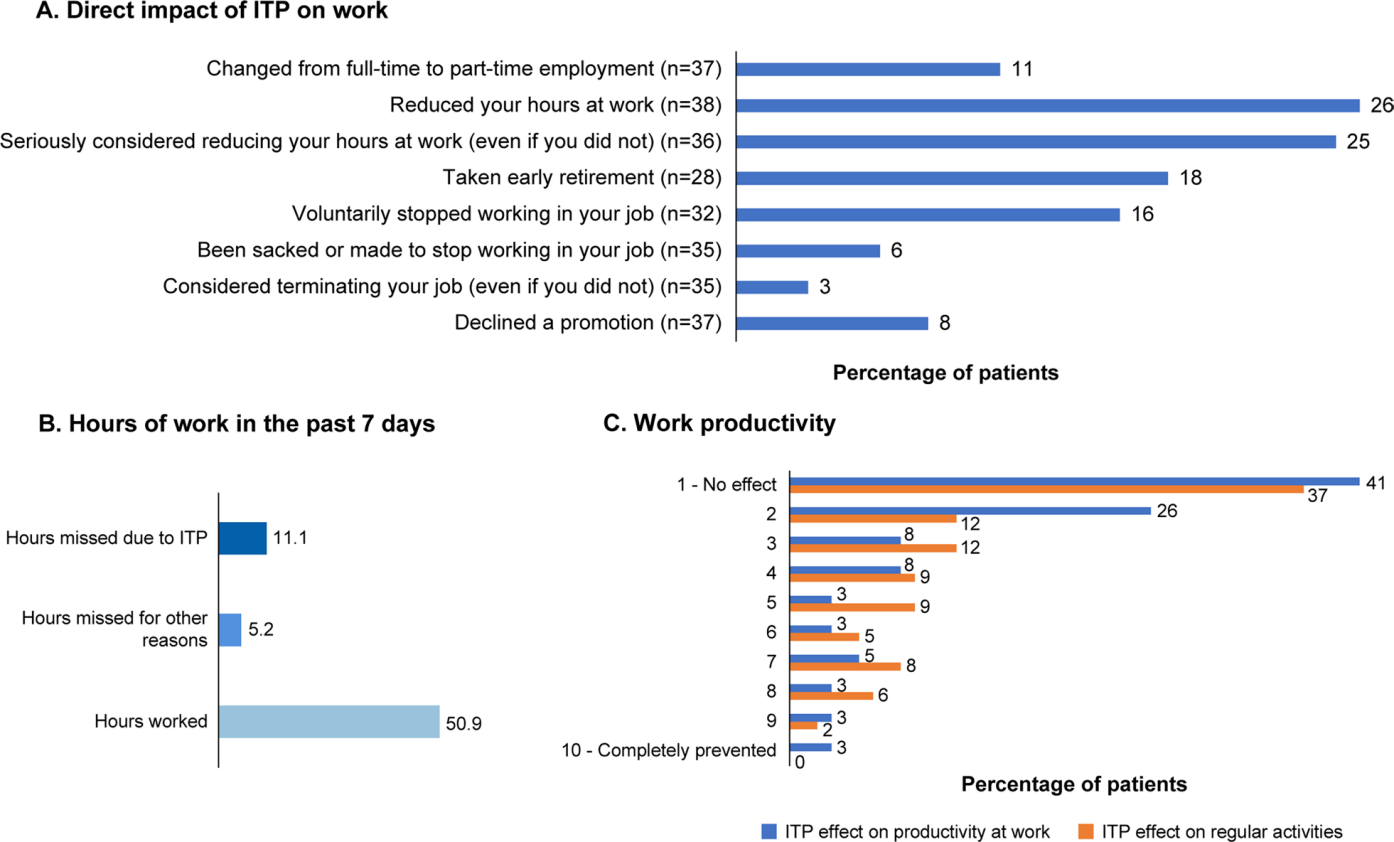
Impact of ITP on work

ITP adversely affected the work and financial situation of patients, with 10 of 38 patients (26%) reducing their work hours and 9 of 36 patients (25%) seriously considering a reduction in their work hours. Patients reported an average of 11.1 h of missed work per week due to the impact of ITP (Fig. 4). The total monthly out-of-pocket expense for a patient with ITP was $211 (~ 16,000 INR), with medicines accounting for more than 60% of this expense ($132 [~ 9600 INR]). Patients also spent an average of 6.1 h/month traveling for their appointments.

Overall, 39 of 65 patients (60%) expressed the need for support (either ‘rarely’, ‘sometimes’, or ‘often’) for an average of 33.7 h/week; homemaking (27 of 39 patients [69%]), transportation (26 of 39 patients [67%]), healthcare (25 of 39 patients [64%]), and management of finances (20 of 39 patients [51%]), were the primary reasons for which support was requested. The key support providers were parents (19 of 39 patients [49%]) and spouses (9 of 39 patients [23%]) (data not shown).

#### Physician

Physicians felt that anxiety about platelet counts and frustrations around having a long-term, rare disease had a severe adverse impact on most of the patients (~ 90%). Daily activities were severely impacted in 4 of 20 patients (21%) and 5 of 20 physicians (25%) felt that ITP had negatively impacted patients’ relationship with their spouses. Overall, interference of ITP in the level of patients’ physical activity was reported as severe by 6 of 20 physicians (30%), and 18 of 20 physicians (90%) felt that ITP greatly impacted patients’ ability to play contact sports or sports with a chance of bleeding injury. A negative impact of ITP on patients’ sex lives (8 of 18 physicians [44%]) and concerns around increased risk of bleeding impacting travel plans (9 of 20 physicians [45%]) was reported by 45% physicians (data not shown).

Almost all physicians (20 of 21 physicians [95%]) did not use any QoL tool, but expressed their desire to use a patient self-assessment questionnaire (12 of 20 physicians [60%] would use it during every consultation, and 7 of 20 physicians [35%] would use it every 6 months). Most physicians (16 of 21 physicians [76%]) expressed that use of a mobile-based app would help in recording patient QoL, while 10 of 21 physicians (48%) expressed that combining paper- and mobile app-based approach would be the preferred method to use. No major differences were observed in the response assessments of physicians based on their workload (data not shown).

### Management of goals and treatment options in ITP

#### Patient

**Fig. 5:**
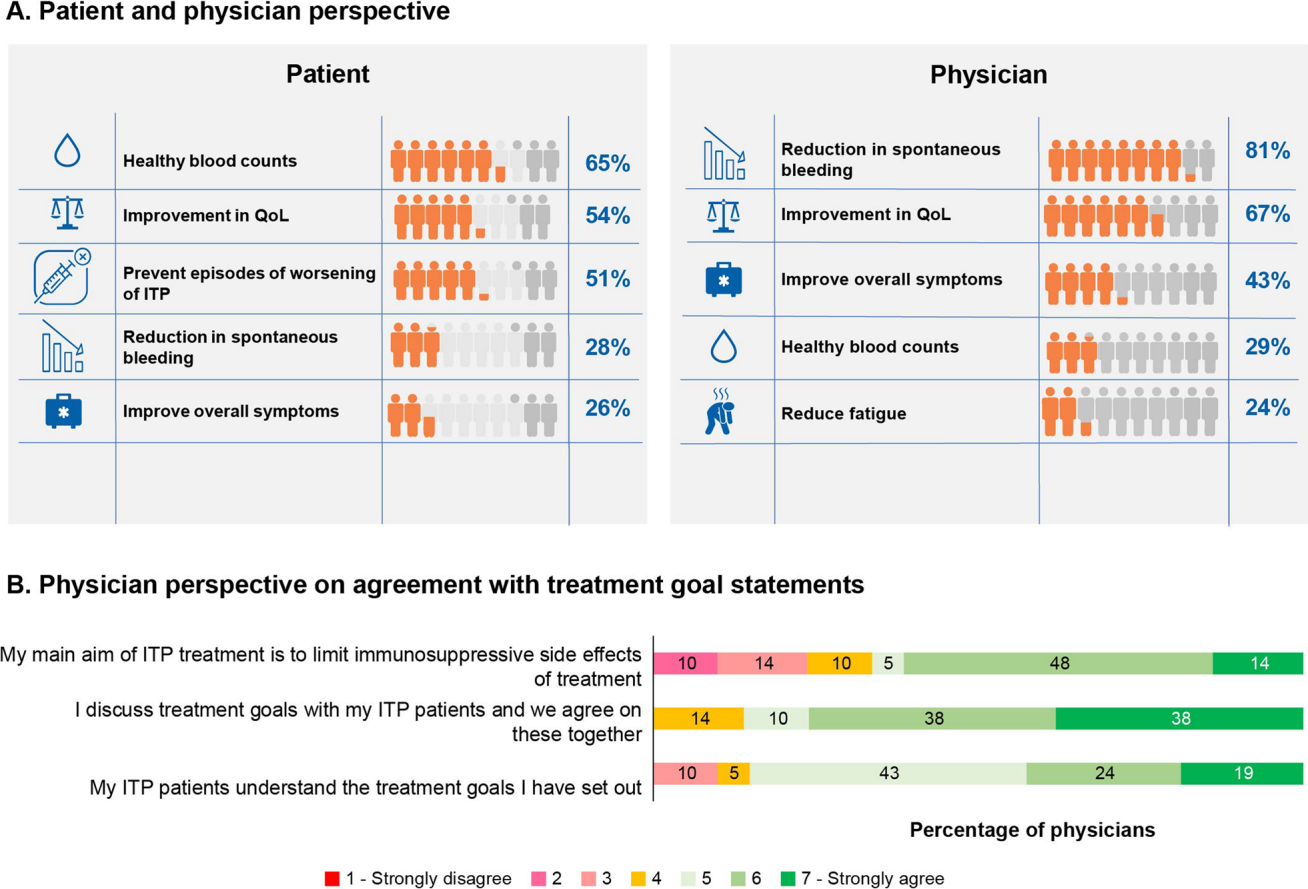
Treatment goals

ITP diagnosis to treatment required an average of 0.9 months, with over half of the patients (34 of 65 patients [52%]) undergoing a period of “wait and watch.” The important treatment goals for patients were healthy blood counts (42 of 65 patients [65%]), improvement in QoL (35 of 65 patients [54%]), prevention of episodes on worsening of ITP (33 of 65 patients [51%]), reduction in spontaneous bleeding (18 of 65 patients [28%]), and an overall improvement in symptoms (17 of 65 patients [26%]) (Fig. 5A). A majority of patients (41 of 65 patients [63%]) strongly agreed that their current treatment was helping them reach their treatment goals.

A once-daily oral pill was preferred by 49 of 65 (75%) patients. At the time of survey completion, the most frequently administered treatments were corticosteroids (38 of 65 patients [58%]), androgens (9 of 65 patients [14%]), anti-CD20 (9 of 65 patients [14%]), thrombopoietin receptor agonists (TPO-RAs; 8 of 65 patients [12%]), and other immunosuppressants (7 of 65 patients [11%]); the average duration of these medications was 4.7 months. When the symptom burden was low, most patients reported undergoing treatment with corticosteroids (27 of 33 patients [82%]), and as the burden increased to moderate and above, corticosteroid use decreased slightly (17 of 24 patients [71%]). The use of androgens (8 of 33 patients [24%] to 11 of 24 patients [46%]), anti-CD20 (9 of 33 patients [27%] to 11 of 24 patients [46%]), and TPO-RAs (3 of 33 patients [9%] to 7 of 24 patients [29%]) increased with increasing symptom burden. Data on treatment satisfaction were based on a low patient number (data not presented here) (data not shown).

#### Physician

Approximately 39% of the newly diagnosed patients were given a trial of observation only. Even among patients who had been previously treated for > 12 months since diagnosis, 30% were put on observation instead of being treated. Splenectomy was considered in 23% of the patients with chronic and recurrent course. Platelet count monitoring was done more routinely in newly diagnosed patients (every 15 days) compared with patients with chronic ITP (every 1.7 months). The major treatment goals for physicians were reduction in spontaneous bleeding (17 of 21 physicians [81%]), better QoL (14 of 21 physicians [67%]), symptom improvement (9 of 21 physicians [43%]), healthy blood counts (6 of 21 physicians [29%]), and reduction in fatigue symptom (5 of 21 physicians [24%]). Nearly 90% of physicians (18 of 21 physicians [86%]) believed that they discussed and agreed on treatment goals with their patients, and 14 of 21 physicians (67%) aimed to limit the immunosuppressive effect of the treatment (Fig. 5B).

**Fig. 6:**
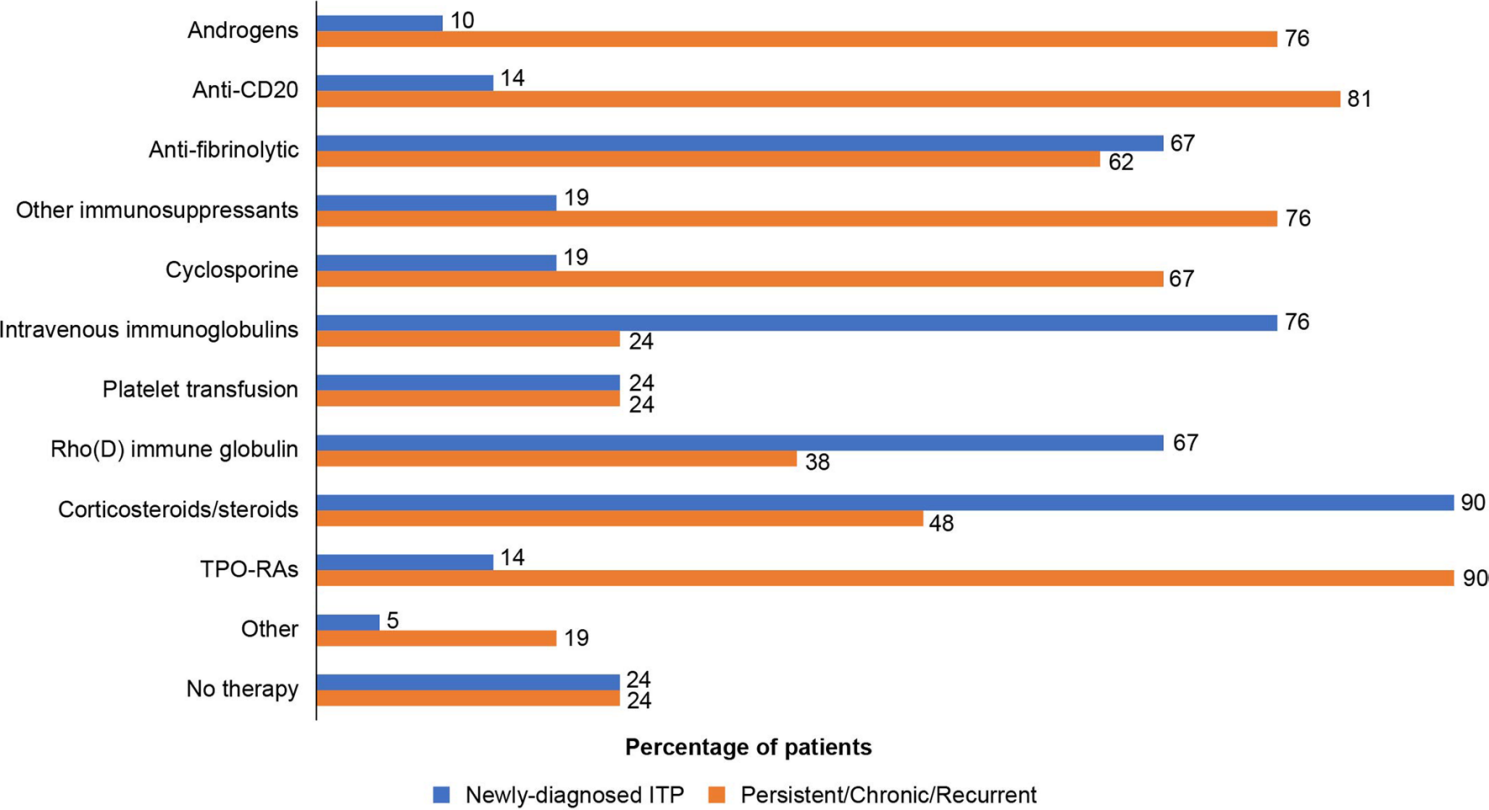
Management of ITP – Prescribed treatments

The most important attributes while making treatment decisions for patients with ITP were offering cure or sustained remission (83%), the ability to reduce bleeding risk (80%), and keeping side effects to a minimum (79%). For both newly diagnosed and chronic ITP, ~ 80% of physicians preferred oral treatment options as the first line of treatment. Corticosteroids (19 of 21 physicians [90%]) and intravenous immunoglobulins (IVIgs; 16 of 21 physicians [76%]) were the preferred treatments in newly diagnosed patients with ITP. TPO-RAs (19 of 21 physicians [90%]) and anti-CD20 (17 of 21 physicians [81%]), followed by androgens (16 of 21 physicians [76%]), were the preferred treatment options in patients with persistent and chronic ITP (Fig. 6; Additional file 3: Figure S3).

For patients relapsing for the first time, corticosteroids (14 of 21 physicians [67%]), followed by androgens (8 of 21 physicians [38%]) and IVIgs/anti-CD20 (7 of 21 physicians [33%] each), were preferred, while during second relapse other immunosuppressants (9 of 21 physicians [43%]) followed by corticosteroids (8 of 21 physicians [38%]) were preferred; by the third relapse, TPO-RAs (11 of 21 physicians [52%]), followed by anti-CD20 therapy (8 of 21 physicians [38%]), were the preferred treatment option (data not shown).

Based on physician perspective, patients treated with TPO-RAs had the least incidence of side effects. A total of 13 of 21 physicians (62%) agreed that they were satisfied with the current treatment options available. Lack of efficacy (21 of 21 physicians [100%]), followed by side effects (19 of 21 physicians [90%]), and cost/coverage (17 of 21 physicians [81%]), was the most important reason for a change in therapy (data not shown).

### Patient and physician relationship

When compared with patients, a lower proportion of physicians were completely satisfied with the various aspects of ITP disease–related care and management (data not shown). While responding to questions on access to information on ITP for their patients, 9 of 21 physicians (43%) expressed that patients faced at least some level of difficulty in accessing information. About half of the physicians indicated that they provided disease management–related information in a leaflet format explaining the contents of the leaflet (9 of 21 physicians [43%]). From the patient perspective, 40 of 65 patients (62%) had not received any information from their HCP. For patients who recieved information from their HCP, it was either through a leaflet (10 of 25 patients [40%]) or through HCPs showing the website content during consultation (8 of 25 patients [32%]) or by being directed to the website for accessing information about the disease (7 of 25 patients [28%]). A large proportion of patients did not have any contact with patient support groups (61 of 65 patients [94%]) (data not shown).

Among 64 of 65 patients (98%) who visited a specialist doctor, an average of 6.5 visits were recorded in the last 12 months, and of these patients, 49 (77%) perceived the frequency of visits to be adequate. None of the patients reported consultation with a psychologist (data not shown).

## Discussion

To the best of our knowledge, this questionnaire-based survey is the first of its kind among patients with ITP and treating physicians in the Indian subcontinent, and provides an insight into the perceptions of both patients and physicians regarding disease diagnosis, signs and symptoms, impact of patient HRQoL, and the approach toward disease management.

A marked difference was observed in the number of patients with ITP seen by physicians in the last 12 months before survey completion between the Indian and global survey data (India: 81, global: 43) [9]. In India, the overall doctor-to-population ratio is 1:1800, which is lower than that the ratio of 1:1000 suggested by ‘High Level Expert Group (HLEG) for Universal Health Coverage’ constituted by the Planning Commission, and endorsed by WHO [7, 13]. Moreover, in India, the population-to-specialist ratio is high [7], which further increases the patient burden of hematologists and hemato-oncologists. It is now widely accepted that ITP is a diagnosis of exclusion and an estimated 15% patients with primary ITP are misdiagnosed (McMaster ITP Registry) [14]. It is also pertinent that the initial diagnosis of ITP is accurate, while avoiding delays based on the challenges associated with patient navigation. Encouragingly, in this survey, the time from symptom presentation to first consultation, and first consultation to diagnosis were both < 1 month based on patients’ response, although from the physicians’ perspective the accuracy of the diagnosis was low, with ~ 25% of physicians estimating that up to 50% patients were misdiagnosed. Even if an accurate diagnosis is made, the high patient burden and the associated low average primary care physician consultation time of ~ 2 min in India [5], physicians tend to primarily treat for bleeding episodes and often underestimate the impact of ITP on QoL. It is therefore imperative that auxiliary healthcare service providers, especially nurses, are trained to assess HRQoL parameters, and along with physicians, adopt app-based or other validated QoL tools for better disease management.

Heavy menstrual bleeding, fatigue, and anxiety surrounding unstable platelet counts were predominantly reported as severe by patients at both diagnosis and survey completion. Physician perspectives on the frequency and/or severity of the most common symptoms and their impact on QoL were not always similar to those reported by patients. While fatigue was reported as severe by ~ 60% of patients at diagnosis, about 33% of physicians perceived it as a symptom that severely affects patient QoL. This trend in underestimation of fatigue by physicians was observed in both the Indian and global data [9]. However, fatigue adversely impacts patient work productivity and social life, and physicians should consider patient-reported fatigue as an important symptom that affects HRQoL [15]. A high frequency of menorrhagia, iron-deficiency anemia, and other nutritional anemias found among Indian patients could be an important contributing factor for fatigue [16–18]. Similar to fatigue events, menorrhagia also impacts a number of HRQoL measures [19, 20] and was reported by a majority of women (> 70%) in this analysis. The fear concerning heavy menstruation could be a major cause of anxiety in most women at the time of ITP diagnosis (based on low-grade evidence) [21, 22]. Of note, anxiety was reported by 43% patients at diagnosis and 35% patients at survey completion. Given that anxiety could be associated with repeated blood count testing, more healthcare visits than required, and changing the consulting physician frequently (doctor shopping), it could result in an overall increase in healthcare cost. Therefore, counselling and participatory medicine is important to ensure a common treatment goal for physicians and patients to address anxiety in ITP. Interestingly, the proportion of patients reporting anxiety as a severe symptom reduced from 61 to 16% from diagnosis to survey completion. This could be partially attributed to the fact that the average disease duration from the time of diagnosis to survey completion was 5.3 years, implying that most patients evaluated in this analysis had chronic ITP. It is often speculated that patients with newly diagnosed ITP have higher anxiety levels due to the uncertainty associated with their disease course [23].

The assessment and improvement of HRQoL parameters generally require a multidimensional approach and should be tailored for the patient, while taking into account the healthcare system, cultural, and economic backgrounds of individual countries [24]. In this subgroup analysis among patients from India, the ILQI questionnaire scores showed that daily life was severely impacted by ITP, with more than half the patients reporting that their work life, education, concentration levels, social lives, and energy levels were negatively affected. In general, the QoL parameters that were highlighted as being a concern include anxiety about platelet counts and frustrations around having a long-term rare disease, high out-of-pocket expenses, inability to perform intense physical exercise or play sports with chances of bleeding injuries, and impact on travel plans due to concerns about increased risk of bleeding and taking medications abroad. The out-of-pocket expenses account for nearly 63% of the total healthcare expenditure in India—one of the highest in the world—reiterating the importance of a country’s healthcare infrastructure in supporting improvement of patients’ HRQoL [24–26]. Although a few public health insurance programs in India cover nonmedical expenditure, such as transportation, lodging, and food costs, for patients and caregivers, there is no provision for incurring the loss of pay suffered by patients or their spouses [26], thereby increasing the socioeconomic burden of the disease. The lack of patient support groups and other professional support for patient counseling add to the emotional burden of ITP in India, as patients almost entirely depend on family, friends, and the treating doctor for support. Patient support groups could not only provide a platform for patients to share their disease experience and provide emotional and moral support but also help educate patients/families, raise public awareness, and aid in raising funds [27]. However, in India, engagement in patient support groups is low. The major constraints in ensuring higher engagement rates could be the lack of awareness, lack of time, or anxiety around discussing the negative aspects of the disease publicly [27]. There is a need to consider a holistic approach toward assessment of symptom burden and impact of ITP on QoL in routine clinical practice in India.

Physician ability to effectively and compassionately communicate the nature of disease and management options is important to build trust in a patient–physician relationship, and shared decision-making is a key element in improving patient–physician communication [28]. Although nearly 90% of physicians included in this survey mentioned that they had included their patients’ perspective during decision-making, the implementation of a participatory decision-making model in ITP, which has been in place for cancer management for a considerable period of time [29], may not be feasible in the Indian context. This could be due to the existing gaps in patient knowledge of the disease and effectiveness of available treatment options [30]. Implementation of a shared-decision model in India needs greater patient education, along with physician awareness and willingness; patient support groups can play a major role in bringing about this change.

A shared-decision model could also help in ensuring that the treatment goals of patients and physicians are completely aligned. Our survey results showed that achievement of healthy blood counts was the most important goal for patients, while for physicians, it was reduction in spontaneous bleeds. Interestingly, improvement in QoL was one of the most important treatment goals for both patients and physicians, underlining the importance of assessing HRQoL among patients with ITP. This was consistent with the global I-WISh data, wherein improvement in QoL was one of the top 3 goals among 38% of patients and 64% of physicians [8, 9].

Overall, the survey data outcomes and driven conclusions must be interpreted with caution, given the small sample size of the respondents, specifically the patient group. Also, this survey did not capture the number of treatment lines received by patients or the remission status. Recall bias and the use of a non-validated HRQoL questionnaire (ILQI) are some of the other limitations of the study. However, the study results need to be considered in the light of the fact that ITP is a rare disease, and currently, in India, there is limited education/awareness among patients regarding the disease.

## Conclusion

Based on the overall respondent assessment, the study highlights the need for education/training of physicians and other healthcare workers on all aspects of ITP disease management—especially fatigue, anxiety, and menorrhagia—and general awareness among physicians and patients on disease management, including treatment goals, and the impact of ITP on QoL. Additionally, it also emphasizes some of the neglected aspects of ITP and provides a good starting point for large-scale future studies in this therapy area.

## Supplementary Information

**Additional file 1. Figure S1**: Symptoms that patients want to be resolved.

**Additional file 2. Figure S2**: Physician perspective on fatigue.

**Additional file 3. Figure S3**: Prescribed treatments based on platelet counts.

## Data Availability

Not applicable.

## Abbreviations

HCP: Healthcare professional
HRQoL: Health-related quality of life
ILQI: ITP Life Quality Index
IRB: Institutional Review Board
ITP: Immune thrombocytopenia
IVIgs: Intravenous immunoglobulins
I-WISh: ITP World Impact Survey
QoL: Quality of life
TPO-RAs: Thrombopoietin receptor agonists
