# Supplementary figures and images for: How do patients and physicians perceive immune thrombocytopenia (ITP) as a disease? Results from Indian analysis of ITP World Impact Survey (I-WISh)
Source: J Patient Rep Outcomes. 2022 Mar 18;6:24. doi: 10.1186/s41687-022-00429-y (PMC8933602; doi:10.1186/s41687-022-00429-y)

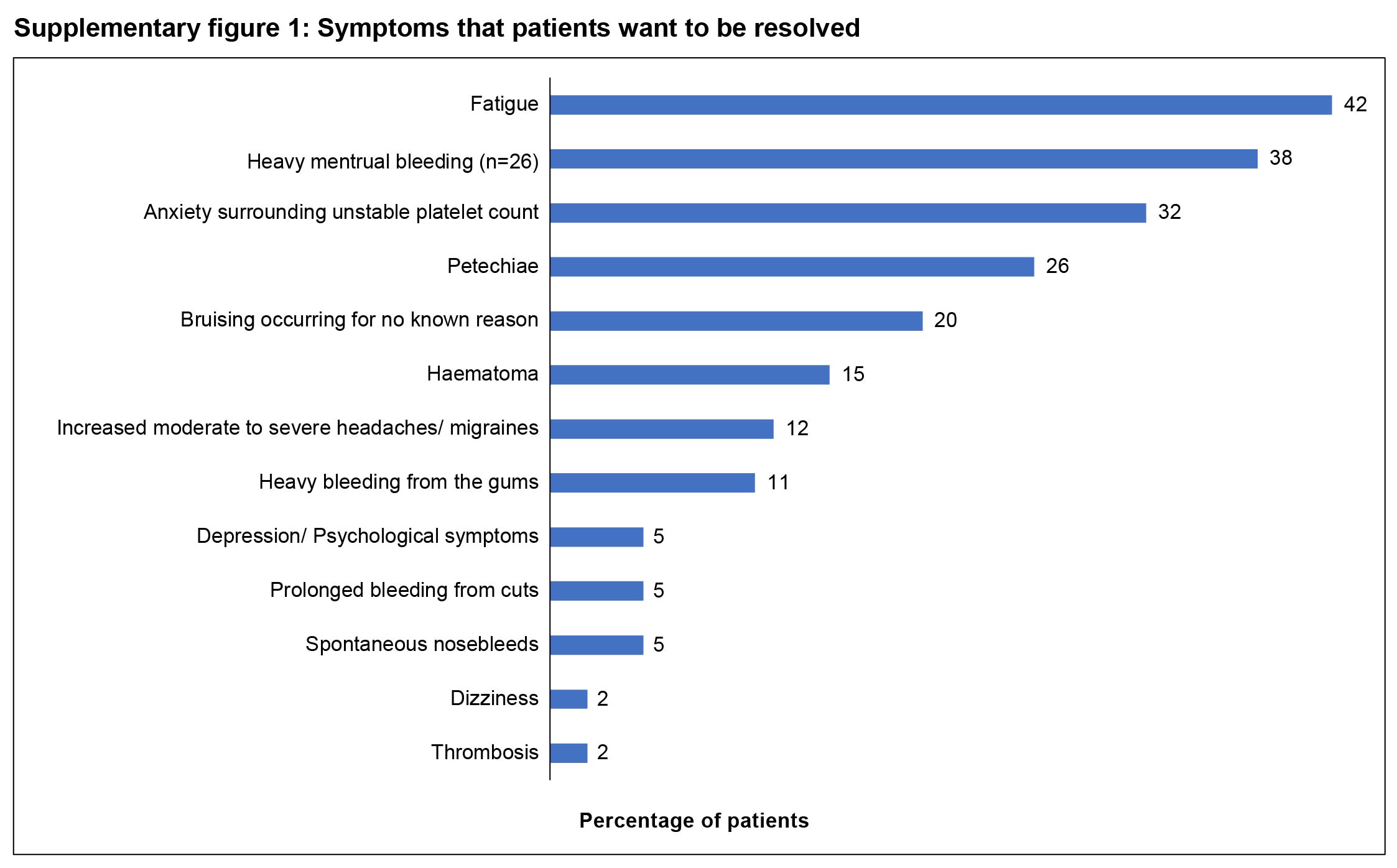

Supplement: Supplementary file 1 — Additional file 1. Figure S1: Symptoms that patients want to be resolved. [file 41687_2022_429_MOESM1_ESM.jpg]

A. Patients with fatigue and severity of fatigue

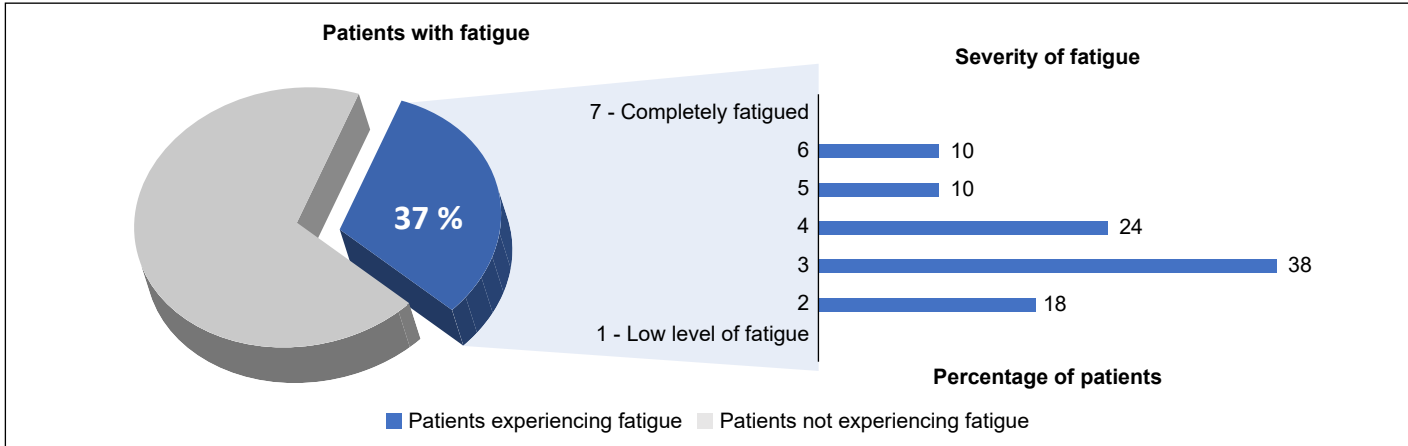

B. Severity of fatigue by platelet count

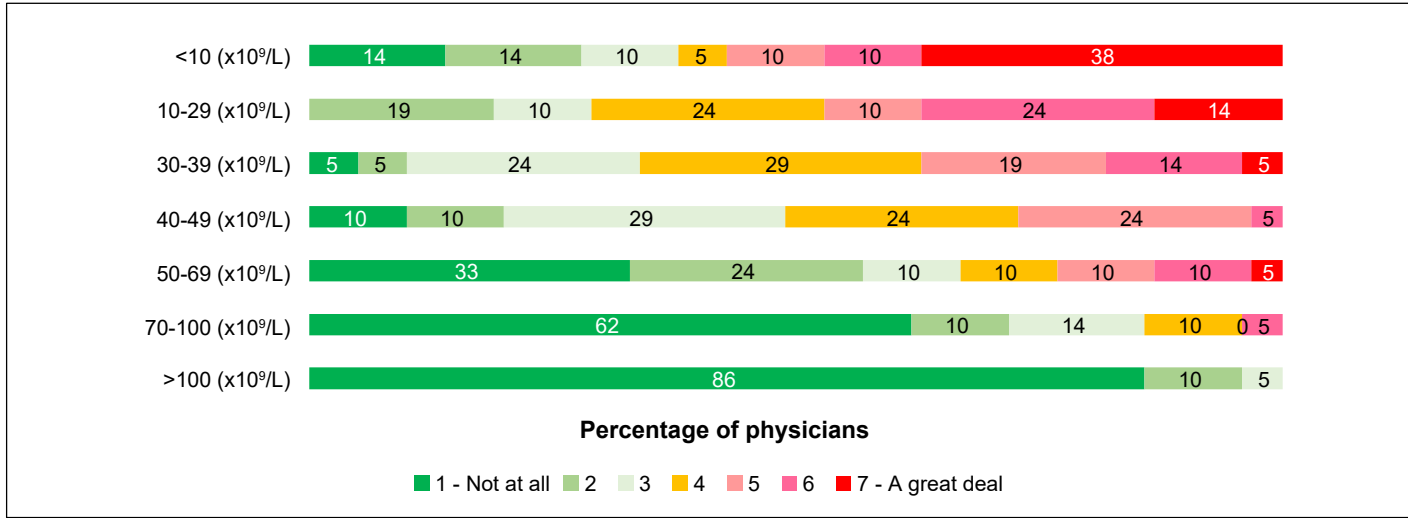

Supplement: Supplementary file 2 — Additional file 2. Figure S2: Physician perspective on fatigue. [file 41687_2022_429_MOESM2_ESM.pdf]

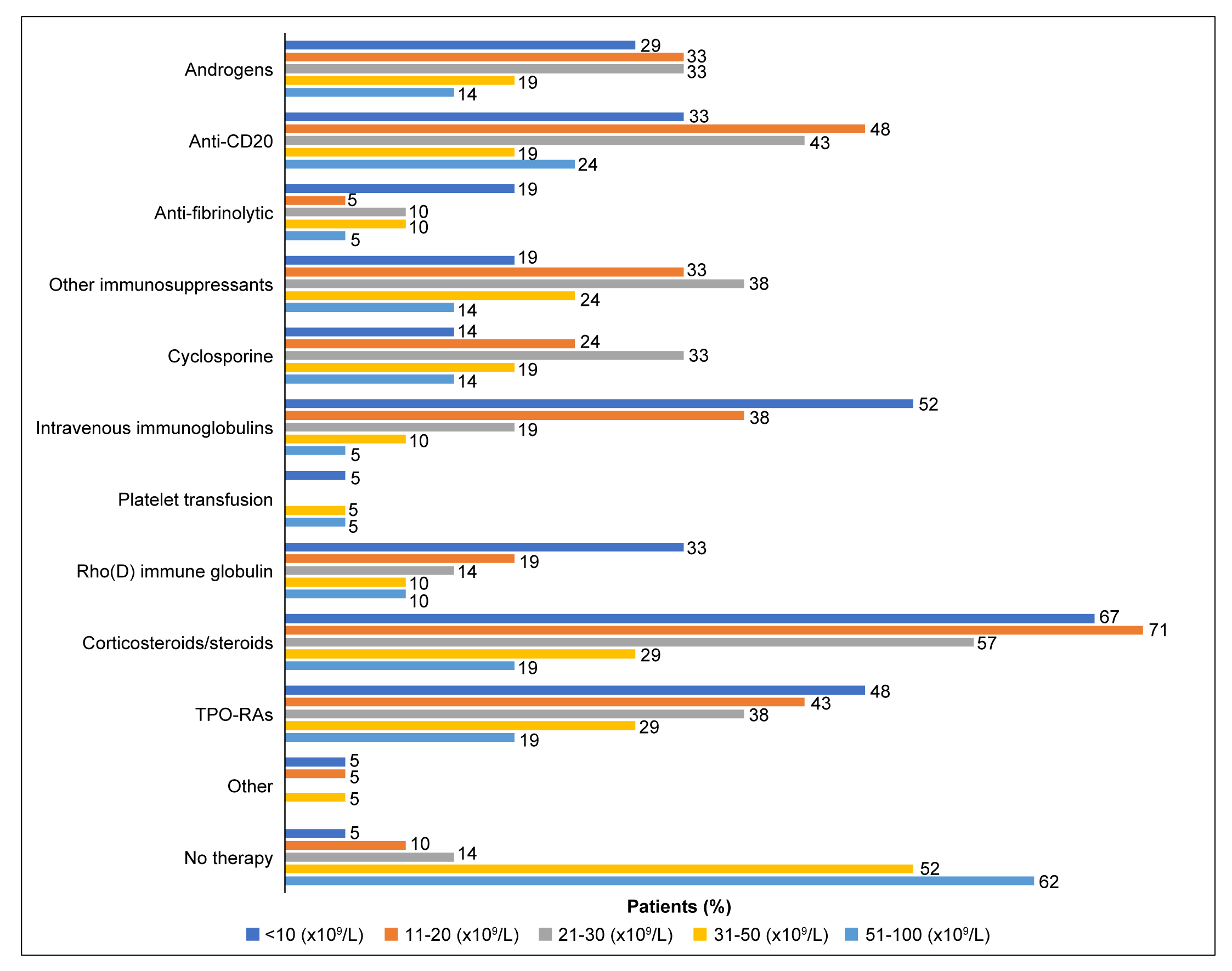

Supplement: Supplementary file 3 — Additional file 3. Figure S3: Prescribed treatments based on platelet counts. [file 41687_2022_429_MOESM3_ESM.tif]
